# Thermal Stability Analysis under Embankment with Asphalt Pavement and Cement Pavement in Permafrost Regions

**DOI:** 10.1155/2013/549623

**Published:** 2013-08-20

**Authors:** Zhang Junwei, Li Jinping, Quan Xiaojuan

**Affiliations:** ^1^School of Civil Engineering and Architecture, Southwest Petroleum University, Chengdu 610500, China; ^2^Key Laboratory of Structure Engineering of Universities in Sichuan, Chengdu 610500, China; ^3^CCCC First Highway Consultants Co., Ltd, Xi'an 710075, China; ^4^School of Civil Engineering, Southwest Jiaotong University, Chengdu 60031, China

## Abstract

The permafrost degradation is the fundamental cause generating embankment diseases and pavement diseases in permafrost region while the permafrost degradation is related with temperature. Based on the field monitoring results of ground temperature along G214 Highway in high temperature permafrost regions, both the ground temperatures in superficial layer and the annual average temperatures under the embankment were discussed, respectively, for concrete pavements and asphalt pavements. The maximum depth of temperature field under the embankment for concrete pavements and asphalt pavements was also studied by using the finite element method. The results of numerical analysis indicate that there were remarkable seasonal differences of the ground temperatures in superficial layer between asphalt pavement and concrete pavement. The maximum influencing depth of temperature field under the permafrost embankment for every pavement was under the depth of 8 m. The thawed cores under both embankments have close relation with the maximum thawed depth, the embankment height, and the service time. The effective measurements will be proposed to keep the thermal stabilities of highway embankment by the results.

## 1. Introduction

Permafrost, a seasonal active layer sensitive to the temperatures, has been attracting deeply more and more researchers in the worldwide because of the permafrost degradation directly threating to the stability of building foundations. In particular, for the high temperature permafrost regions, all kinds of pavement diseases of the embankment would appear easily if the constructed embankments were not properly protected before. For example, there is the unevenly transverse settlement of pavement due to the asymmetric distribution of the thermal field under the permafrost embankment. For this reason, the thermal stability of cement concrete pavement in permafrost regions has been always studied [1–4]. The thermal state difference between south faced slope and north faced slope of embankment is considered as the most important factor to cause the unevenly transverse settlement of pavement. As a result, the thermal state difference may result in asymmetric thermal regime in embankment. And the unevenly transverse deformation may appear thereupon. In opinions of Yu [5], the permafrost degradation is mainly caused by the natural thermal equilibrium destroyed under permafrost embankment [6–11]. Affected by global warming and embankment construction disturbances, the heat exchange between atmosphere and ground surface had been changed after highway pavements were built in the permafrost regions. The permafrost degradation acceleration begins to grow due to the heat exchange condition changing. In particular, for warm permafrost regions, all kinds of pavement diseases would appear easily because of the acceleration of permafrost degradation if the constructed embankments were not properly protected. The previous studies on the acceleration of permafrost degradation indicate that embankment diseases and pavement diseases are closely related to thermal stability under permafrost embankment in the permafrost regions. It is a key factor to keep the embankment thermal stability to solve the problems of embankment diseases and pavement diseases. The unevenly distribution of the thermal field under the permafrost embankment is the direct reason of the embankment deformation in permafrost regions of the Qinghai-Tibetan Plateau. The maximum deformation of highway permafrost roadbed lies off the center of the embankment [5, 12]. 

To thoroughly understand the failure mechanisms of the Qinghai-Tibetan highway embankment in the permafrost regions, radiation and thermal balance were observed on the surface of asphalt pavement. And these observation stations were located on ground surfaces of different types of permafrost embankments between the Kunlun Pass and the No.66 station port. The calculated results based on the observation suppose that the part of heat influencing significantly on the heat regime of the embankment is considered as the main reason of the Qinghai-Tibetan highway embankment failure. And the part of heat was responsible for the formation of a thawed core below the embankment in the high-temperature permafrost section [13]. From the geohazard investigations of the embankments along the Qinghai-Tibet Railway, permafrost degradation results in some main geohazards such as thawing settlement, frost-heave, and freezing-thawing induced hazards. All of them might potentially influence the embankment stability including settlement, burying, and laterally thermal erosion [3]. The thermal stability of the permafrost embankment will highly have been changed because thermal effect problems associated with slope orientations result in the maximum thawed depth position being deviated in the roadbed transverse direction rather than thawed on the embankment central line [4]. The sunny-shady slope seriously impacts on the thermal and deformation stability of the highway embankment in warm permafrost region by analyzing both the observational geotemperature and deformation data of the embankment in the experimental section K369+100 along the National Highway 214 on Qinghai-Tibet Plateau [14]. From the insitu geothermal data of up to 15 years of 5 typical sections along the Qinghai-Tibet Highway, embankment settlement is closely related to the process of degrading of underling permafrost extensively in the five typical sections with different characteristics. With the increase in the mean annual ground temperature, the thawing rate firstly increases and then fluctuates as temperature rise rate increases and then decreases at the mean annual ground temperature of about −0.5°C [15]. Different from the previous study, in cold seasons, the temperature under the reinforced concrete component was higher than the shoulders by analyzing temperature characteristics of tested embankments at the Changchun site of Harbin to Dalian Passenger Dedicated Line. This difference decreases with the depth of roadbed. In warm seasons, these phenomena appear as a reverse trend, and also the temperature difference decreases with the depth of roadbed as usual. In different parts of the roadbed, the maximum seasonal frozen depths were all higher than the natural ground. The reason is that the roadbed materials changed the heat exchange process between the air and the ground surface [16]. Thus thaw settlement is the main embankments distresses of highway in permafrost regions, according to survey data of the Qinghai-Tibet Highway. It can be effectively mitigated or even controlled by raising the embankment height [17].

It is found from the aforementioned studies that cement concrete pavement and asphalt concrete pavement are presently two common pavement structures used in permafrost embankment engineering. Black asphalt pavement has strong temperature susceptibility. And it can absorb more solar radiation than cement concrete pavement. So the black asphalt pavement has a higher road surface temperature. This will seriously affect the temperature field and the stability of permafrost under the asphalt concrete pavement embankment. The aforementioned studies only consider the temperature field under asphalt pavement permafrost embankment with strong temperature susceptibility. Although some measures were used to deal with the embankment diseases and the pavement diseases, the disease problems could not be solved fundamentally. The most important reason is that the thermal stability under the permafrost embankment has not been quite figured out. As a result, cement concrete pavement begins to be selected to use to keep the temperature field of embankment stable instead of black asphalt pavement in the permafrost regions. Because the cement concrete pavement is seldom used under the natural conditions in the permafrost regions until now, it is not almost seen on the theory of cement concrete pavement or embankment diseases. In addition, these diseases are closely related with not only pavement structures but also the temperature field under both permafrost embankments. Therefore, the finite element method will be applied to calculate temperature field under both permafrost embankments with adjustable embankment height. The temperature field changes between asphalt pavement and cement concrete pavement will be analyzed at different embankment width. The studying results will supply reliable theory for the designing of the embankment stability. They are in favor of sustainable development of permafrost engineering because of keeping the thermal stability under the embankment by taking effective use of natural ventilation.

## 2. Analysis of the Shallow Ground Temperature

**Figure 1:**
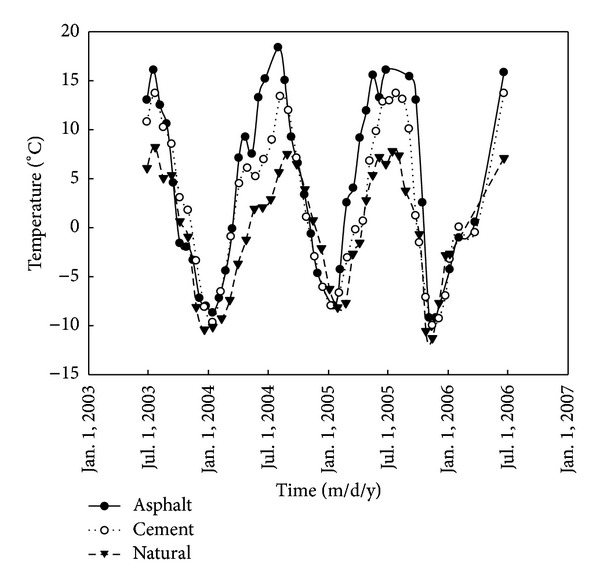
The ground temperature at the depth of 0.5 m versus time.

The cement section of K374+975 and the asphalt section of K375+300 are the borders upon sections. The ground temperature of the both pavements has being observed since July 2003 Figure 1 shows that ground temperature at the depth of 0.5 m versus. time under the three pavements types from August 1, 2003 to August 1, 2006 according to the observation results of the ground temperature. These observation results were mainly obtained through the various drill-holes by the geological drilling. And we can find that there is an obvious ground temperature difference between the section of K374+975 and the section of K375+300. As is shown in Figure 1, the construction of both the cement pavement and the asphalt pavement caused a large change in the shallow thermal regime partly. There is also distinct difference in the ground temperature at the depth of 0.5 m between both pavements. The ground temperature under cement pavement is always lower than that under asphalt pavement. And there is distinctly seasonal difference in the ground temperature at the depth of 0.5 m between them. It is obviously larger in summer than in winter. In summer, the ground temperature under the cement pavement is higher 5-6°C than that under the natural ground at the depth of 0.5 m. However, the ground temperature under the asphalt pavement is even higher 10°C than that under the natural ground at the depth of 0.5 m. In winter, the difference between them is little. Both of them are less than 1.0°C. The reason is that the temperature sensitivity of the asphalt is higher than that of the cement.

In order to further narrate the ground temperature difference between both pavements, Table 1 gives the annual average temperature of 2003–2006 years under asphalt pavement in the section K417+970 of seasonally frozen ground and cement pavement in the section K418+030 of permafrost, as well as the difference between them at the depths of 0.5–2.0 m. It is seen from Table 1 that the annual average temperature under asphalt pavement in the section K417+970 of seasonally frozen ground at the depths of 0.5–2.0 m is higher (1.84–2.53°C) than that under cement pavement in the section K418+030 of permafrost from 2004 to 2006. The ground temperature difference between both pavements in the section of seasonally frozen ground is even larger than that in the section of permafrost. It is also shown that constructed cement pavement is more stable than constructed asphalt pavement in the thermal stability of embankment.

As can be seen from Table 2, the ground temperature at the depth of 2.0 m under the asphalt pavement is 0.47–0.92°C higher that than under cement pavement. This result manifests good thermal stability of permafrost under cement pavement. There is also a temperature difference between the asphalt pavement and the cement pavement at the same depth under the pavement. And the difference reduces gradually with the increment of the depth.

It can be found from Table 3 that the ground temperature of the embankment centre at the depth of 2.0 m under the asphalt pavement is 0.68–1.34°C higher than that under cement pavement. The ground temperature of the embankment centre at the depth of 2.0 m–8.0 m under the asphalt pavement is 0.06–0.29°C higher than that of cement pavement. But the temperature difference began to become less and less beyond 8 m under the ground surface. This result manifests that for the embankment with a width of 8.5 m, 8 m is the maximum depth of the pavement inflecting on embankment. Otherwise, the ground temperature under the embankment is influenced little on beyond 8 m.

**Figure 2:**
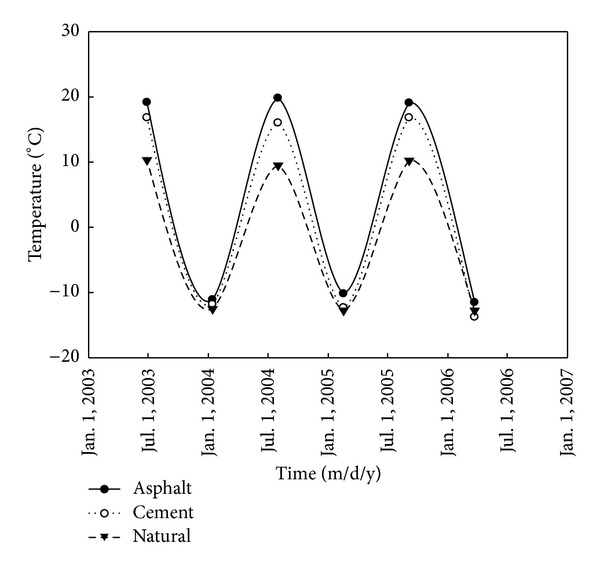
The curve of the surface temperature amplitude under different pavements.

Both the most high ground temperate and the lowest ground temperature at the depth of 2.0 m under the pavement in Table 4 are from the section K374+975 and the section K375+300 in the national highway 214 for the natural ground, cement pavement, and asphalt pavement, respectively. But the curve of the surface temperature amplitude under different pavements in Figure 2 is not based on Table 4.

## 3. Finite Element Analysis Model

### 3.1. Mathematical Model

The freezing and thawing cycle of permafrost embankments is the process of heat and mass transfer accompanying heat flow and redistribution so the mechanism of the internal water and heat function of the embankment in the permafrost regions may be considered to be a heat transfer considering moisture migration accompanying phase changes.

The freeze-thaw cycle in permafrost embankment is accompanied by the redistribution of temperature field and moisture migration. The mechanism of water and thermal in the permafrost embankment can be attributed to the thermal conduction problems accompanying phase changes. The following hypotheses are presented for the selected mathematical model considering the phase changes of freeze-thaw which influences the temperature field and seepage field of the permafrost embankment.

1. Layers soil of each embankment section is homogeneous.
2. No external load acts on soil layer during freezing and thawing.

Because thermal conduction term is far greater than convection term in the freeze-thaw process of frozen soil, the effect of the convection, mass transfer, the latent heat of vaporization, and chemical potential; are negligible in calculating analysis compared to the heat diffusion and the heat diffusion equation only considers soil skeleton and thermal conductivity of water, so the ice-water phase change may be written as follows

(1) $$\begin{array}{c} \frac{\partial }{\partial x}\left(k_{x}\frac{\partial T}{\partial x}\right)+\frac{\partial }{\partial y}\left(k_{y}\frac{\partial T}{\partial y}\right)=C\rho \frac{\partial T}{\partial t}-L\rho_{i}\frac{\partial W_{i}}{\partial t}, \end{array}$$

where *T* is transient temperature and *t* is time. *k*_*x*_ and *k*_*y*_ are components of soil equivalent thermal conductivity. *C*, *ρ*, *L*, *W*, and *W*_*i*_ are soil equivalent volumetric heat capacity, soil density, latent heat freezing and thawing of water, soil moisture, and unfrozen soil moisture, respectively. And *x* and *y* represent horizontal direction and vertical direction respectively in the cross section of the embankment.

### 3.2. Computational Region

**Figure 3:**
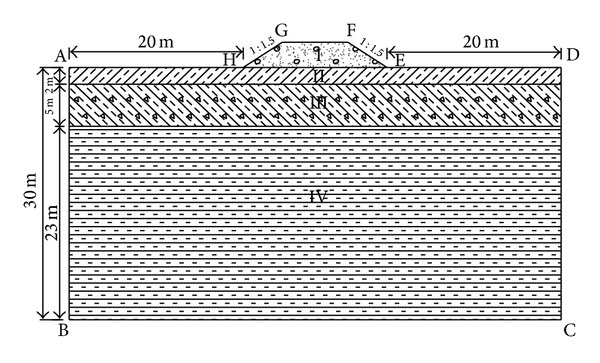
The computational region. Zone I is gravel backfill, zone II is sub-clay, zone III is crushed rock and sub-clay, and zone IV is argillaceous rocks.

Figure 3 shows the computational region. Three embankments widths of 8.5 m, 12.0 m, and 22.5 m, representing the highways with different grades (People's Republic of China Profession Standards, 1998), were selected. The gradient of the embankment slope is 1 : 1.5. The embankment height is adjustable ranging from 1 m to 3 m with a step of 1 m. The flank fields of both sides are 20 m from the foot of slope, and the lower boundary is 30 m below the natural ground surface. The thermal stability of permafrost embankment under asphalt concrete pavement and cement concrete pavement is analyzed with different heights under every embankment width.

The most major difference that permafrost has from other soils is that its property has close relationship with temperature. The heat capacity of the frozen soil skeleton only considers the volumetric heat capacity of freezing mode and thawing mode in computation. In addition, the thermal conductivity value only considers the effect of freeze-thaw state while ignoring the effect of temperature. Soil parameters within computational region are listed in Table 5 [3, 10].

### 3.3. The Boundary Conditions

The lower boundary temperature condition is determined by the secular measured ground temperature gradient at the depth of 30 m in Plateau permafrost region. The temperature gradient can be described as follows:

(2) ∂T∂y=0.02 C°m.

The temperature gradient of the temperature boundary condition of embankment is 0 in the horizontal direction due to the lateral natural ground of embankment away from the embankment. Consider the following equation:

(3) $$\begin{array}{c} \frac{\partial T}{\partial x}=0. \end{array}$$

As the temperature boundary condition values of embankment slope are slightly lower than the upper temperature boundary condition values of cement concrete pavement, the upper temperature boundary condition of cement concrete pavement is simplified for the following trigonometric functions:

(4) $$\begin{array}{c} T=T_{0}+R_{0}t+A\sin\left(\frac{2\pi t}{365}+B\right), \end{array}$$

where *T*_0_ is the initial annual ground temperature distribution of the embankment surface, *t* is operating time, *A* is temperature amplitude of the embankment surface, *R*_0_ is increasing rate of ground surface temperature caused by the global climate warming, *R*_0_ = 0.02 °C/a, *B* is the initial calculated phase, and *A* and *T*_0_ are obtained by analyzing the measured temperature of Zuimatan testing segment of the national highway 214 in Table 6.

## 4. Computational Results and Analysis

### 4.1. Modeling Verification

**Figure 4:**
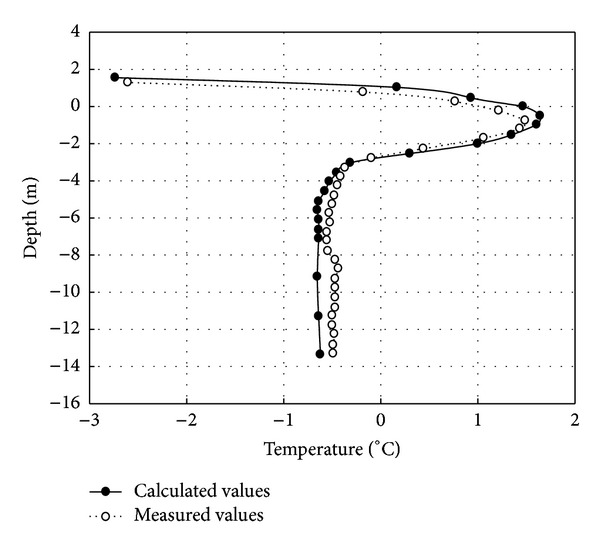
Measured and calculated temperature values at the maximum thawed depth of embankment center (1 year after the construction of cement concrete pavement).

**Figure 5:**
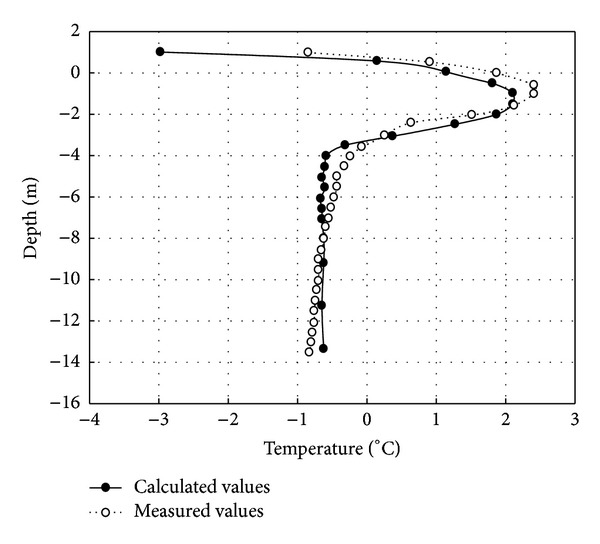
Measured and calculated temperature values at the maximum thawed depth of embankment center a year after the construction.

According to the aforementioned boundary conditions, the initial conditions, and the thermal physical parameter of the unstable high temperature permafrost with an average annual temperature of −3.5°C, GEO-SLOPE is used separately to calculate the maximum thawed depth under the center line of cement concrete pavement in Figure 4 and asphalt pavement in Figure 5 one year after embankment constructed. As can be seen in Figures 2 and 3, the ground temperature curves separately calculated by the GEO-SLOPE are basically consistent with the measured temperature curve under cement concrete pavement and asphalt pavement respectively. Compared respectively to the measured temperature values of the K422+820 section of Zuimatan testing segment along the national highway 214 for cement concrete pavement and asphalt pavement, the maximum thawed depth under the permafrost embankment happened in November of 2004, that is when the 2nd year after Zuimatan testing segment construction of the national highway 214 was completed. The consistency between the calculated temperature curve and the measured temperature curve verifies the reliability of the mathematical model simultaneously.

### 4.2. Results Analysis

**Figure 6:**
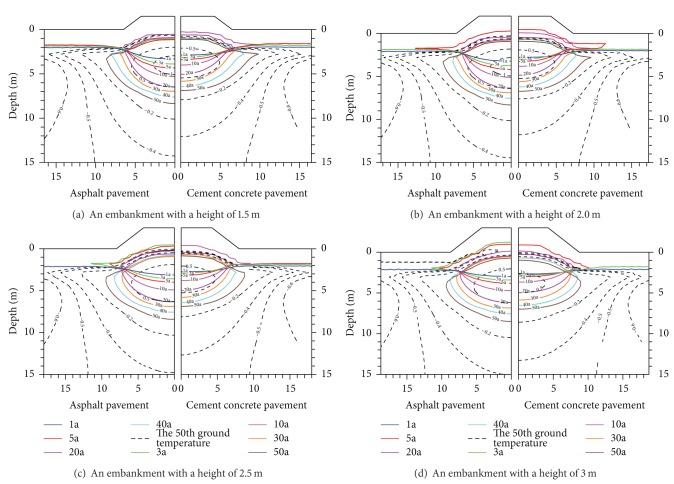
Variations of the maximum thawed depth and temperature value of the 50th year under concrete pavement and asphalt pavement (width of 8.5 m).

*The Thawing Core Generating*. The temperature field under cement concrete pavement embankment and asphalt pavement embankment is firstly analyzed separately at a pavement width of 8.5 m. As can be seen in Figure 6, the residual thawed layers appeared under the embankments with a height of 1.5 m, 2.0 m, 2.5 m, and 3.0 m, respectively. The reason is that the temperature rises gradually after the two embankments constructed completely. The maximum thawed depth under permafrost embankments when the residual layers begin to appear is considered as the maximum thawed depth of permafrost embankments. The appearance time of the residual thawed layers has close relationship with pavement type, pavement height, and pavement width. Figure 6 also shows the maximum thawed depth calculated under the two pavement structures 1a, 5a, 10a, 20a, 30a, 40a, and 50a (a representing year), respectively, after they were completely built. The 50th year temperature field under the two pavement structures is also seen from Figure 6. For the pavement width of 8.5 m, the maximum thawed depth under asphalt pavement has been always greater than cement concrete pavement every year from the first year to the 30th year after the embankments were completely built. The maximum thawed depth differences between the two pavements become greater and greater as the embankment height decreases.

Figure 6 shows that for asphalt pavement permafrost embankment and cement concrete pavement embankment, the maximum thawed depth differences between the both pavements structures present on decreasing tendency at the same embankment width as the embankment height increases. And the maximum thawed depth of the cement concrete pavement embankment is always less than asphalt pavement permafrost embankment at the same service time. Embankment height and embankment thermal resistance are considered as the main factors resulting in the maximum thawed depth differences. The external factors affect the temperature field under permafrost embankment through the pavement. The embankment thermal resistance is increasing gradually with the increase of the embankment height. The external factors influence less and less the temperature field of permafrost embankment through the pavement as the embankment height increases. The different material performances of pavement structures will generate different thermal resistances. Therefore, the degree of the external factors affecting the temperature field under permafrost embankment through the pavement will present differences because of the pavement material performances. For example, when the embankment height is 1.5 m, the 50th year maximum thawed depth under cement concrete pavement after embankment begins to operate is basically as much as the 10th year maximum thawed depth under asphalt pavement. However, the maximum thawed depth differences between both pavements become little as the embankment height increases although the 50th year maximum thawed depth under cement the concrete pavement after embankment begins to operate is greater than the 30th year one under the asphalt pavement. It can be concluded from the previous analysis that the thermal stability of permafrost under cement concrete pavement is obviously better than asphalt pavement at the same service time if taking the same lower embankment height.

It is found from Figure 6 that for cement concrete pavement and asphalt pavement, respectively, the time of the maximum thawed depth developing the fastest under every pavement appears in the first 20 years after the highways begin to operate at different embankment height. The developing rate of the maximum thawed depth under every pavement slows down significantly after 20 years of operation. The change is mainly caused by the instability of the temperature field under permafrost embankments disturbed by human activities and engineering construction after the highway embankment has been built. The temperature field of permafrost embankments begins to become extremely unstable when the maximum thawed depth increases. But after a long operation, the degree that the maximum thawed depth under every pavement is influenced little by external temperature, climatic conditions, and engineering construction gradually decreases. In this case, the temperature field under permafrost embankment tends to stabilize. And the developing rate of the maximum thawed depth shows the characteristics of first increasing and then decreasing with the embankment operation time increasing. In addition, the time when the thawing core appears under asphalt pavement permafrost embankment is significantly earlier than cement concrete pavement during the operation time when the embankment height is 1.5 m, 2.0 m, 2.5 m, and 3.0 m, respectively. To every embankment, the range of pavement type affecting the temperature field under permafrost embankment is approximately within 5 m distant from outside embankment slope when the width of the two embankments is 8.5 m. The range and degree of the pavement material types influencing the temperature field of permafrost embankment are closely related to the height of embankment. But in general, the range of pavement type influencing the temperature field under permafrost embankment presents a decreasing trend with the increment of the embankment height.

To the permafrost embankment with a width of 8.5 m, the range of pavement type influencing on the temperature field under permafrost embankment is approximately within 5 m distant from outside embankment slope. And the pavement types have no influences on the temperature field under permafrost embankment outside 5 m distant from outside embankment slope. The range and degree that the pavement types influence on the temperature field under permafrost embankment have close relations with the height of embankment. But in general, the range of pavement type influence on the temperature field under permafrost embankment presents decreasing trend with the increment of the embankment height.

From the analysis of the previously calculated results, we can find that the maximum thawed depth under the cement concrete pavement permafrost embankment has increased not obviously with the highway permafrost embankment service time increasing compared to the asphalt pavement although the maximum thawed depth of cement concrete pavement has increased with the highway permafrost embankment service time increasing.

**Figure 7:**
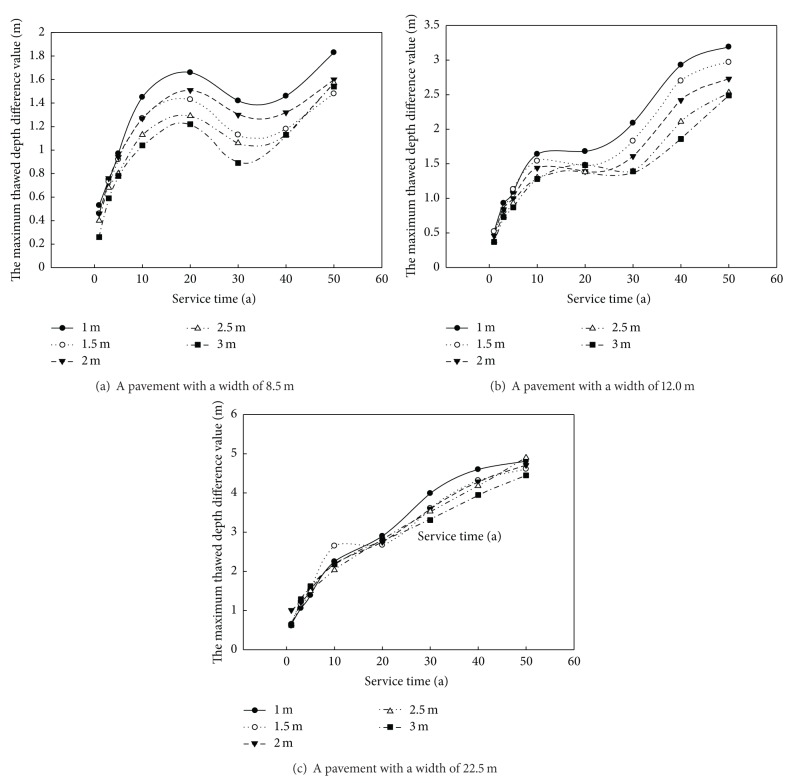
The maximum thawed depth difference value under concrete pavements and asphalt pavements versus service time curve under different pavement width.

In order to clearly describe that the thermal stability of permafrost under cement concrete pavement is better than that under asphalt pavement, Figure 7 shows the relationship between the maximum thawed depth under the cement concrete pavement and service time and the different embankment height. The maximum thawed depth differences under the asphalt pavement and cement concrete pavement under different pavement widths in the high ice content permafrost region are with an average temperature of −3.5°C. The relationship between the maximum thawed depth under the cement concrete pavement and the service time varies because of the different width of cement concrete pavement and asphalt pavement. For example, the maximum thawed depth differences under both pavements with a pavement width of 8.5 m increase with the increment of service time and then tend to decrease a certain time after operation. But when the pavement width of the two pavements is 12 m and 22.5 m, respectively, the relationship between the maximum thawed depth differences under the two pavements and service time approximately present on nolinear increasing. It also shows that the pavement width greatly has influences on the change rate of the maximum thawed depth difference. The maximum thawed depth differences under two pavements increase apparently with the increment of service time when the pavement width of the embankment becomes larger. It also shows that the larger width of the cement concrete pavement is an advantage to the thermal stability under the cement concrete pavement.

**Figure 8:**
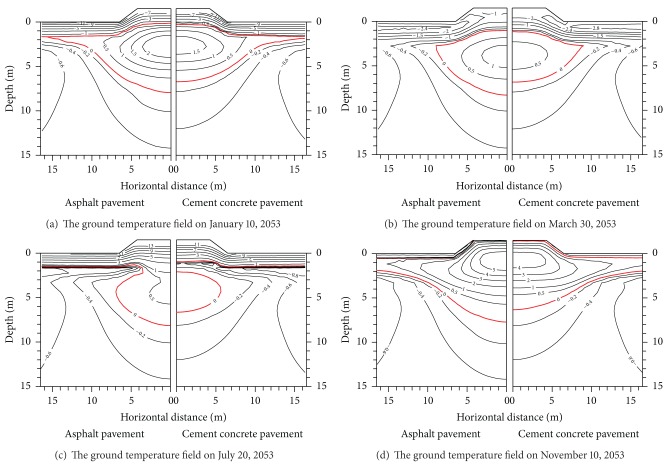
Temperature values of the 50th year under concrete pavement and asphalt pavement (width of 8.5 m).

**Figure 9:**
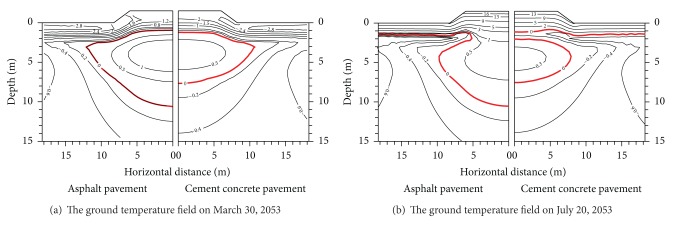
Temperature values of the 50th year under concrete pavement and asphalt pavement (width of 12.0 m).

*The Temperature Differences under Different Pavement Types*. The temperature field under every pavement with the same embankment height presents different characteristics under different time or different embankment width. We can find that the 50th year maximum thawed depth under each pavement changes with different reasons in Figures 8 and 9 when the embankment width is 8.5 m.

Figure 8 shows the 50th year ground temperature field under both pavements with a width of 1.5 m after the embankment service. It is seen from Figure 8 that January 10 is the lowest temperature, March 30 is the maximum thawed depth, July 20 is the maximum air temperature, and November 10 is in freezing period. In freezing period (January 10), there exists a close thawed core that is higher than 0°C under each pavement. The ground temperature curves are parallel to the embankment surface profile above the close thawed core. The ground temperature and ground temperature gradients between pavement and plane at 1.5 m depth are both apparently higher than the ones between natural ground and plane at 1.5 m depth. On the contrary, the ground temperature curves have large differences under the close thawed core. The differences are induced by pavement material performances. The shallow ground temperature under every pavement begins to rise when the maximum thawed depth appears under both embankments On March 30, 2053. The differences between the shallow ground temperatures also begin to increase gradually. Comprised with the ground temperature field in freezing period, the ground temperature field differences are not apparent. When the ground temperature reaches the highest temperature on July 20, 2053, the isothermal curve of the shallow ground is basically parallel to the embankment surface profile. But the shallow ground temperature under the asphalt pavement is higher 2.0°C than that of the concrete pavement. There is a more semienclosed thawed zone under the asphalt pavement. And there is a close thawed zone (thawed core) under the concrete pavement. The ground temperature in the thawed core of the concrete pavement is lower than that of the asphalt pavement. This is mainly caused by the concrete pavement having stronger temperature susceptibility than the black asphalt pavement. From November 10, the shallow ground temperature under every pavement begins to decrease. The maximum thawed depth in the embankment center is far greater than the natural ground. But the maximum thawed depth in the embankment center is still less than the artificial one. Although the natural ground begins to freeze, the time of the asphalt pavement refreezing is apparently later than that of the concrete pavement.

**Figure 10:**
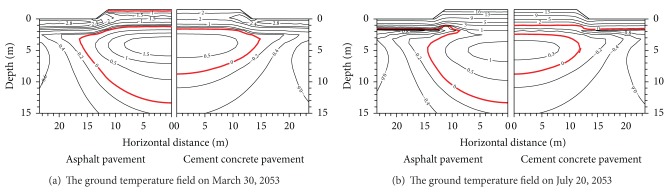
Temperature values of the 50th year under concrete pavement and asphalt pavement (width of 21.5 m).

Figures 9 and 10 show that for the embankments with widths of 12.0 m and 21.5 m, there are big differences at the ground temperature under pavements. The ground temperature curves are basically identical for the embankments with widths of 8.5 m, 12.0 m, and 21.5 m. This indicates that pavement materials influence the difference distribution. Particularly in summer, the shallow ground temperature differences are very obvious between the concrete pavement and the asphalt pavement. There are small shallow ground temperature differences between the concrete pavement and the asphalt pavement. This proves further that the concrete pavement has stronger temperature susceptibility than the black asphalt pavement.

It is seen from Figures 9 and 10 that in different seasons, pavement materials greatly have influence on the ground temperature under the embankment. On the contrary, pavement widths influence less the ground temperature under the embankment. Therefore, the ground temperature change under the embankment by the pavement material is more than that by the pavement widths. In addition, the influence area of the temperature field under both the asphalt pavement and the concrete pavement is distant 5 m or so from the embankment slope. Outside 5 m distance from the embankment slope, the influence area of the temperature field under both the asphalt pavement and the concrete pavement becomes less. The influence area has begun to decrease with the increment of embankment height. 

## 5. Conclusions

From the aforementioned results and analyses, we can find several significant conclusions for the thermal stability analysis under embankment with asphalt pavement and cement pavement in permafrost region.

1. There is a ground temperature difference between asphalt pavement and concrete pavement at the same depth within 8 m under pavement. The ground temperature difference changes with the seasons. The difference is 2.2–5.2°C in summer while the difference is basically less than 1°C in winter.
2. The maximum thawed depth difference between asphalt pavement and cement pavement decreases with the increment of the embankment height. The development rate of the maximum thawed depth begins to increase with the embankment height decreasing. The time when the development rate of the maximum thawed depth appears is the first 20 years after the embankments operate. From then on, the development rate of the maximum thawed depth begins to decrease.
3. The temperature under cement concrete pavement is always lower than that of asphalt pavement at the same service time in the computational region. To the permafrost embankment with the same width, the maximum thawed depth under asphalt pavement is greater than that of cement concrete pavement. The maximum thawed depth difference between the two pavement structures becomes more significant with the embankment width increasing.
4. The developing rate of the maximum thawed depth presents the characteristic of first increasing and then decreasing under different embankment height. The appearance time of the thawing core under asphalt pavement is earlier than that of cement concrete pavement.
5. In different seasons, pavement materials greatly influence the ground temperature under the embankment. On the contrary, pavement widths influence less the ground temperature under the embankment. Therefore, the ground temperature change under the embankment by the pavement material is more than that by the pavement widths. In addition, the influence area of the temperature field under both the asphalt pavement and the concrete pavement is distant 5 m or so from the embankment slope. Outside 5 m distance from the embankment slope, the influence area of the temperature field under both the asphalt pavement, and the concrete pavement becomes less. The influence area has begun to decrease with the increment of embankment height.
6. The maximum thawed depth differences under the two pavement structures present the trend of decreasing with the service time increasing a certain time after the pavements begin to operate if the pavement widths are less. The maximum thawed depth differences between the two pavement structures approximately present linearity with operating time if the pavement widths are greater. The maximum thawed depth under cement concrete pavement is always less than asphalt pavement.
7. It is more important to assure the sustainability of the embankment engineering in the high temperature permafrost region, and the effective measures must be taken to protect permafrost because of the extremely fragile ecological environment.

## Figures and Tables

**Table 1:** The ground temperatures difference under asphalt pavement and cement pavement (the depth of 0.5 m).

Time	Ground temperature difference between asphalt and natural pavements/°C	Ground temperature difference between asphalt and cement pavements/°C
2004	3.93	1.84
2005	4.43	2.36
2006	3.71	2.03
The average temperature of 03–06 years	4.02	2.06
The average temperature Zhu Linnan supplied	4.0	2.0

**Table 2:** Comparisons of the ground temperature at the depth of 2.0 under pavement.

Depth/m	Asphalt pavement The average temperature of 03–06 years/°C	Cement pavement The average temperature of 03–06 years/°C	Ground temperature difference/°C
0.5	2.69	1.77	0.92
1.0	2.50	1.60	0.90
1.5	2.05	1.43	0.62
2.0	1.61	1.14	0.47

**Table 3:** The average temperatures of 03–06 years under asphalt pavement and cement pavement.

Depth/m	Cement pavement/°C			Cement pavement/°C			Ground temperature difference/°C
	Center hole	Natural hole	Temperature increment magnitude	Center hole	Natural hole	Temperature increment magnitude	
−1.3	2.00	/	2.00	3.26	/	3.26	1.26
−0.8	2.11	/	2.11	3.45	/	3.45	1.34
−0.3	1.82	/	1.82	2.89	/	2.89	1.07
0.2	1.59	/	1.59	2.27	/	2.27	0.68
0.7	1.15	−0.53	1.68	1.65	−0.32	1.97	0.29
1.2	0.72	−0.35	1.07	1.02	−0.32	1.34	0.27
2.2	0.10	−0.47	0.57	0.14	−0.67	0.81	0.24
3.2	−0.20	−0.57	0.37	−0.19	−0.68	0.49	0.12
4.2	−0.30	−0.58	0.28	−0.35	−0.71	0.36	0.08
5.2	−0.36	−0.59	0.23	−0.45	−0.75	0.30	0.07
6.2	−0.40	−0.59	0.19	−0.53	−0.78	0.25	0.06
7.2	−0.42	−0.60	0.18	−0.59	−0.80	0.21	0.03
8.7	−0.46	−0.59	0.13	−0.67	−0.82	0.15	0.02

**Table 4:** The temperature of linear regression in surface of natural ground, asphalt pavement, and cement pavement.

Monitoring section	Pavement type	Monitoring contents	04	05	06
K374+975	Natural ground	The maximum temperature	11.22	10.32	10.87
		The minimum temperature	−12.85	−12.77	−13.31
		The annual average ground temperature	12.03	11.54	12.09
		The average temperature amplitude of 04–06 years/°C	11.89		

K374+975	Cement pavement	The maximum temperature	16.82	16.22	16.71
		The minimum temperature	−11.60	−12.44	−13.54
		The annual average ground temperature	14.21	14.33	15.12
		The average temperature amplitude of 04–06 years/°C	14.55		

K375+300	Asphalt pavement	The maximum temperature	19.17	19.79	19.32
		The minimum temperature	−11.02	−10.17	−11.41
		The annual average ground temperature	15.09	14.98	15.36
		The average temperature amplitude of 04–06 years/°C	15.15		

**Table 5:** Soil parameters used in finite element analysis.

The thermal parameters	*λ*_*f*_/(kJ/(m · °C · d))	*λ*_*u*_/(kJ/(m · °C · d))	*C*_*f*_/(kJ/(m^3^ · °C))	*C*_*u*_/(kJ/(m^3^ · °C))	Volumetric water content
Gravel backfill	129.60	120.96	1 827	2 226	0.08
Subclay	155.52	129.60	2 066	2 718	0.30
Rock and subclay	190.00	86.40	2 468	3 806	0.50
Argillaceous rocks	228.40	160.10	2 594	3 892	0.15

*λ*_*f*_ and *λ*_*u*_ are the thermal conductivity coefficients of frozen soil and unfrozen soil, respectively. *C*_*f*_ and *C*_*u*_ are the volumetric heat capacity coefficients of frozen soil and unfrozen soil, respectively.

**Table 6:** The annual ground temperature and the ground temperature amplitude in the top boundaries.

Pavement type	*T*_0_/°C	The ground temperature amplitude *A*/°C
Natural ground surface/°C	−1.0	11.89
Asphalt pavement/°C	3.0	15.15
Cement pavement/°C	1.0	14.55
The gravel slope/°C	0.2	14.2
